# Understand spiciness: mechanism of TRPV1 channel activation by capsaicin

**DOI:** 10.1007/s13238-016-0353-7

**Published:** 2017-01-02

**Authors:** Fan Yang, Jie Zheng

**Affiliations:** Department of Physiology and Membrane Biology, University of California, Davis, CA 95616 USA

**Keywords:** capsaicin, TRPV1, ligand gating, cryo-EM, computation, spiciness

## Abstract

Capsaicin in chili peppers bestows the sensation of spiciness. Since the discovery of its receptor, transient receptor potential vanilloid 1 (TRPV1) ion channel, how capsaicin activates this channel has been under extensive investigation using a variety of experimental techniques including mutagenesis, patch-clamp recording, crystallography, cryo-electron microscopy, computational docking and molecular dynamic simulation. A framework of how capsaicin binds and activates TRPV1 has started to merge: capsaicin binds to a pocket formed by the channel’s transmembrane segments, where it takes a “tail-up, head-down” configuration. Binding is mediated by both hydrogen bonds and van der Waals interactions. Upon binding, capsaicin stabilizes the open state of TRPV1 by “pull-and-contact” with the S4-S5 linker. Understanding the ligand-host interaction will greatly facilitate pharmaceutical efforts to develop novel analgesics targeting TRPV1.

## Introduction

**Figure 1:**
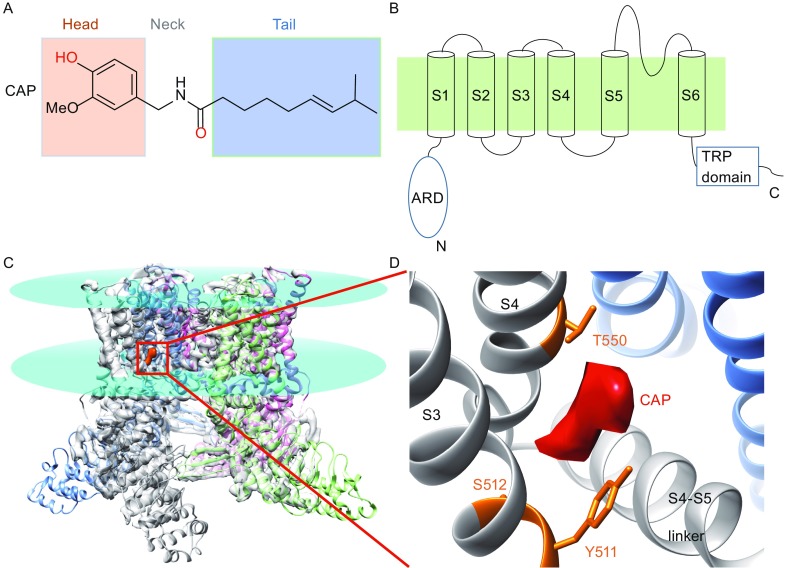
**Capsaicin and TRPV1**. (A) Chemical structure of capsaicin. The vanillyl Head and hydrophobic Tail groups are shaded in orange and blue, respectively. The atoms forming hydrogen bonds with TRPV1 are highlighted in red. (B) Schematic diagram showing the topology of a TRPV1 subunit. Membrane is shaded in green. (C) High resolution structure of rat TRPV1 determined by cryo-EM (atomic model: 3J5R in PDB; electron density map, 5777 in EMD). It is clear that capsaicin (electron density boxed by solid line) binds to the transmembrane domains. Lipid membrane boundaries are indicated by cyan disks. (D) A zoom-in view of the capsaicin binding pocket. Residue important for capsaicin activation identified by mutagenesis and functional studies are colored in orange. The electron density of capsaicin is colored in red

Many people over the world enjoy spiciness in foods. Indeed, spicy hot pot is a signature dish in southwest China and chili peppers are essential ingredients in Mexican cuisine. Many health benefits are believed to originate from chili pepper consumption (Szallasi and Blumberg, 1999). However, we humans are the only species that deliberately seeks spicy foods (Nilius and Appendino, 2013), while most animals are repelled by the irritating sensation. Plants of the genus *Capsicum,* family *Solanaceae* such as chili peppers are the most common source of spiciness, as their fruits contain a group of pungent molecules named capsaicinoids. Among the capsaicinoids, capsaicin ((*E*)-*N*-[(4-Hydroxy-3-methoxyphenyl)methyl]-8-methylnon-6-enamide) is the most abundant in quantity, though not much spicier than other capsaicinoids such as dihydrocapsaicin, homocapsaicin and homodihydrocapsaicin based on the Scoville scale (Scoville, 1912). Capsaicin was first isolated from paprika and cayenne in the late 19^th^ century (Thresh, 1876), with its chemical structure reported in 1923 (Nelson and Dawson, 1923). Similar to other capsaicinoids, capsaicin contains a vanillyl group (which we refer to as the Head), an amide group (the Neck) and a fatty acid chain (the Tail) (Fig. 1A).

To elicit the spicy sensation, capsaicin has long been known to excite nociceptive neurons by increasing their membrane permeability to cations (Bevan and Szolcsanyi, 1990; Oh et al., 1996). Based on structure-activity relationship studies of capsaicin and its derivatives (Szolcsanyi and Jancso-Gabor, 1975; Szolcsanyi and Jancso-Gabor, 1976), as well as the dose-dependent and saturable nature of capsaicin activation (Szallasi, 1994), the existence of a capsaicin receptor has been predicted in the 1990s. Indeed, such a receptor for capsaicin was cloned from rat dorsal root ganglia in 1997 (Caterina et al., 1997). This receptor was originally known as vanilloid receptor 1 (VR1), and later formally named as transient receptor potential vanilloid 1 (TRPV1) (Montell et al., 2002). TRPV1 is a tetrameric channel with both N and C termini of each subunit located intracellularly (Fig. 1B) (Caterina et al., 1997). Though the capsaicin receptor is known to function as TRPV1 homotetramer, different subunits within the TRP family are able to form heteromeric channels (Cheng et al., 2007, 2010; Fischer et al., 2014), with the heteromeric channels exhibiting distinct functional properties as compared to the homomeric channels (Cheng et al., 2012). The transmembrane core region of TRPV1, containing six transmembrane helices per subunit (S1 to S6), exhibits the same topology and many structural features as voltage-gated potassium channels (Zheng and Ma, 2014). TRPV1 is a non-selective cation channel; when it is activated by capsaicin, sodium and calcium ions flowing through TRPV1 into the cell to depolarize nociceptive neurons, leading to action potential firing and finally the sensation of spiciness (Caterina et al., 1997).

Besides capsaicin, TRPV1 can be activated by many physical and chemical stimuli such as noxious heat (Caterina et al., 1997), low extracellular pH (Tominaga et al., 1998), divalent cations such as Mg^2+^ and Ba^2+^ (Yang et al., 2014; Cao et al., 2014; Ahern et al., 2005), as well as animal toxins (Bohlen et al., 2010; Siemens et al., 2006; Yang et al., 2015). These stimuli are allosterically coupled to the close-to-open transition of TRPV1 (Matta and Ahern, 2007; Diaz-Franulic et al., 2016; Zheng, 2013) so distinct activation pathways exist for specific stimuli. For instance, proton and peptide toxins bind to extracellular pore domain of the channel, while capsaicin binds to its transmembrane domains. Sensitivity to a wide spectrum of physical and chemical inputs allows TRPV1 to serve as a polymodal sensor for noxious stimuli (Zheng, 2013). Consistent with the nociceptive role, TRPV1 knock-out mice exhibit impaired sensation to thermal-mechanical acute pain (Caterina et al., 2000).

While acting as a polymodal receptor, TRPV1 nevertheless shows exquisite sensitivity and selectivity for capsaicin. The EC_50_ value of capsaicin activation is in sub-micromolar range (Caterina et al., 1997; Yang et al., 2015), while the maximum channel open probability attained at saturating concentrations can reach close to unity (Yang et al., 2015; Cui et al., 2012; Hui et al., 2003). Minor modifications of capsaicin molecule can drastically reduce its potency, or even turn it into an effective antagonist (Appendino et al., 2003; Appendino et al., 2005). Interestingly, while being a potent activator, capsaicin also exhibits high selectivity for TRPV1 as it does not activate other homologous channels within the TRPV family (Yang et al., 2016). Beyond this family, capsaicin has been implied to inhibit signal transducer and activator of transcription 3 (Bhutani et al., 2007) and carbonic anhydrase 2 (Ye et al., 2015), though the concentrations required for the inhibitory effects are at least 10 times higher than what is required for activating TRPV1. Substantial work has been put into exploiting the potent and selective TRPV1 activation by capsaicin for pharmaceutical purposes (Szallasi and Blumberg, 1999; Lazar et al., 2009). Understanding the mechanism of TRPV1 activation by capsaicin is thus greatly needed to guide pharmaceutical efforts. This review focuses on the rapid progress in this area.

## Early mutagenesis and functional studies

Capsaicin activation of TRPV1 was first investigated by making chimeras between capsaicin sensitive and insensitive orthologs. Like rodent TRPV1, avian TRPV1 is also activated by noxious temperature (Jordt and Julius, 2002). However, as birds are able to ingest plants rich in capsaicin (which is beneficial to the plants as birds can help disperse their seeds), their TRPV1 should be less sensitive to capsaicin. Indeed, in 2002, the chicken TRPV1 was demonstrated to be insensitive to capsaicin up to 100 µmol/L (Jordt and Julius, 2002). When different domains of rat and chicken TRPV1 were swapped, chimeras containing the transmembrane segments 2 to 4 of rat TRPV1 and the rest of chicken TRPV1 exhibited sensitivity to vanilloids like capsaicin and resiniferatoxin. This observation suggested that the S2 to S4 segments are key to capsaicin binding, while the remaining gating machinery required for capsaicin activation is preserved in both channels. Furthermore, point mutations S512Y and Y511A at the intracellular end of S3 on rat TRPV1 were able to eliminate capsaicin sensitivity, suggesting capsaicin may bind to the vicinity of these residues (Jordt and Julius, 2002). Nonetheless, both S512 and Y511 are conserved between rat and chicken TRPV1; therefore, these two residues alone cannot explain the difference in capsaicin sensitivity between rat and chicken TRPV1. This chimera/mutation study illustrated the importance of transmembrane segments in capsaicin activation, however, many critical questions such as the location of capsaicin binding site had not been completely answered.

Like chicken TRPV1, rabbit TRPV1 exhibits much reduced capsaicin sensitivity (Gavva et al., 2004). A study published in 2004 showed that, when the S3 to S4 segments of rat TRPV1 were transferred to rabbit TRPV1, high capsaicin sensitivity can be transferred as well. More strikingly, substantial increase in capsaicin sensitivity could also be achieved when a single residue I550 on S4 of rabbit TRPV1 was mutated to its rat counterpart: a threonine (Gavva et al., 2004). From these two early studies using chicken and rabbit TRPV1, we have learnt that Y511, S512 and T550 are critical residues for capsaicin activation. While it was speculated that these residues may participate in capsaicin binding, a definitive answer on how they exert their impact remains elusive for another decade.

Single-channel recording has been a valuable technique to study ligand-host interaction in ion channels (Zheng and Trudeau, 2015), as it reveals the functional states and microscopic transitions that are hidden from macroscopic current recordings (Sakmann and Neher, 2009). For example, the ligand binding and gating kinetics of acetylcholine receptors have been well characterized with single-channel recordings (Purohit et al., 2007; Grosman et al., 2000). This technique has also been applied to study other ligand-gated channels such as cyclic nucleotide-gating channels (Sunderman and Zagotta, 1999a, b), large-conductance calcium-activated potassium channels (Piskorowski and Aldrich, 2002), and another TRP channel, transient receptor potential melastatin 8 channel (Fernandez et al., 2011). Based on single-channel recordings, a study published in 2003 suggested that capsaicin binding stabilizes the channels at high open probabilities (Hui et al., 2003). Together with mutagenesis tests, these early studies had laid a solid foundation to understand how capsaicin activates TRPV1, while the detailed structural basis of the ligand activation process was not understood until the first high-resolution structures of TRPV1 were resolved by cryo-EM.

## Early structural studies of TRPV1

3D structures of an ion channel in the absence and presence of ligands play a crucial role in the study of ligand gating process. For instance, the crystal structure of the C terminus in hyperpolarization-activated, cyclic nucleotide-modulated (HCN) channel greatly contributed to understanding how its ligands such as cAMP and cGMP bind and gate this channel (Zagotta et al., 2003). As for TRPV1, due to technical difficulties, for a long time the only available high-resolution structure was limited to its N terminus Ankyrin-repeat like domain (ARD) reported in 2007 (Lishko et al., 2007). ARD binds ATP and modulates calcium-dependent channel desensitization upon capsaicin activation. ARDs of the homologous TRPV2 (Jin et al., 2006), TRPV3 (Shi et al., 2013), TRPV4 (Inada et al., 2012) and TRPV6 (Phelps et al., 2008) were also revolved by crystallography. In addition, a short piece of the TRPV1 C terminus was resolved by crystallography as an α-helix in the presence of calmodulin (Lau et al., 2012). However, without a view of the whole channel, the knowledge we learned from these structures regarding capsaicin activation was very limited.

The structure of full-length TRPV1 by cryo-EM was first reported in 2008 (Moiseenkova-Bell et al., 2008). However, with a low resolution at 19 Å only the subunits arrangement, as well as the relative orientation between transmembrane and intracellular domains were clearly discernable. Around the same time, TRPV4 (Shigematsu et al., 2010) and TRPC3 (Mio et al., 2007) structures were also studied with cryo-EM. Again only 35 Å (TRPV4) and 15 Å (TRPC3) resolutions were achieved. The low resolution of these structures, due to technical difficulties faced by the cryo-EM method at that time, limited insights that can be gained regarding the channel architecture themselves and, for TRPV1, the mechanism of capsaicin activation.

As an alternative approach, TRPV1 structure was modeled computationally. Using voltage-gated potassium channel structures as homology templates, either the pore domain (Ferrer-Montiel et al., 2004) or full-length TRPV1 (Fernandez-Ballester and Ferrer-Montiel, 2008) was modeled. TRPV1 transmembrane domains were also modeled using a voltage-gated sodium channel as the template (Yang et al., 2013). As the sequence identities between TRPV1 and the template channels (less than 20%) are rather low, and there is a lack of experimental constraints for model building, limited insights were gained from those studies.

## Structural revelation of capsaicin binding site

The breakthrough for TRPV1 structural biology occurred in 2013. With the development of direct-detection camera and better algorisms dealing with sample movements and image processing, TRPV1 channel structure in the closed state was determined by cryo-EM at an astonishing resolution of 3.4 Å (Liao et al., 2013). At this resolution, not only the secondary structures but also some of the residue side-chains were clearly observed, which allowed the *de novo* building of atomic model of TRPV1 (Fig. 1C). Furthermore, two open states of TRPV1 were determined at atomic resolution with either capsaicin or resiniferatoxin/double knot toxin (Cao et al., 2013). These cryo-EM structures unequivocally pin-pointed the location of capsaicin-binding pocket, which is formed by S3, S4 and S4-S5 linker within the membrane (Fig. 1C and 1D). This is in close agreement with mutagenesis and functional studies: residues Y511, S512 and T550 identified in these studies locate right around the pocket (Jordt and Julius, 2002; Gavva et al., 2004), and dramatic perturbations to intracellular structures often leave capsaicin activation undisturbed (Ma et al., 2016). More interestingly, a small electron density was observed inside the capsaicin-binding pocket in the capsaicin-bound structure. This provided so far the most direct evidence of the location of bound capsaicin. Interestingly, in the closed (apo) state of TRPV1, an electron density was also observed, which was interpreted to indicate that this pocket may be occupied by a lipid molecule in the absence of capsaicin. Therefore, capsaicin may have to compete with such a lipid molecule in order to bind and activate TRPV1. In addition, by comparing the closed state and open state, a slight outward movement of the S4-S5 linker, away from the central ion conducting pore, was observed upon capsaicin binding. Since the S4-S5 linker in voltage-gated channels couples movements of the S4 voltage sensor to the S6 activation gate (Lu et al., 2002; Yarov-Yarovoy et al., 2012), such a movement of the S4-S5 linker in TRPV1 may underline how capsaicin binding leads to channel opening.

The success of cryo-EM study of TRPV1 structure has marked a new era of structural biology for membrane proteins. For TRP channels, structures of TRPV2 (Huynh et al., 2016; Zubcevic et al., 2016) and TRPA1 (Paulsen et al., 2015) were soon determined by cryo-EM at atomic resolutions, while TRPV6 structure was resolved by crystallography (Saotome et al., 2016). However, a high-resolution structure does not solve all the problems. For instance, though the capsaicin-bound open state of TRPV1 was determined, the capsaicin molecule was registered within the binding pocket as an electron density much smaller than its chemical structure (Fig. 1D) (Cao et al., 2013), hence it remained unclear how capsaicin is orientated. There was also no information on how capsaicin interacts with channel protein, as the resolution of this region is only about 4.5 Å. Recently, with the lipid nanodisc technique TRPV1 structure was determined at 2.9 Å (Gao et al., 2016). At this improved resolution, the binding orientations of several TRPV1 modulators such as double-knot toxin and resiniferatoxin were clearly defined. A few structured lipid molecules interacting with the channel were also resolved. However, no capsaicin bound state was resolved in that study. A likely interpretation is that parts of the bound capsaicin and RTX retain substantial mobility, which makes it hard for structural investigation alone to reveal the molecular details of ligand-channel interaction. Therefore, cryo-EM structures are not the end of story but rather a solid foundation for follow-up studies to understand how capsaicin activates TRPV1.

## Hybrid approaches in the post-structural era

**Figure 2:**
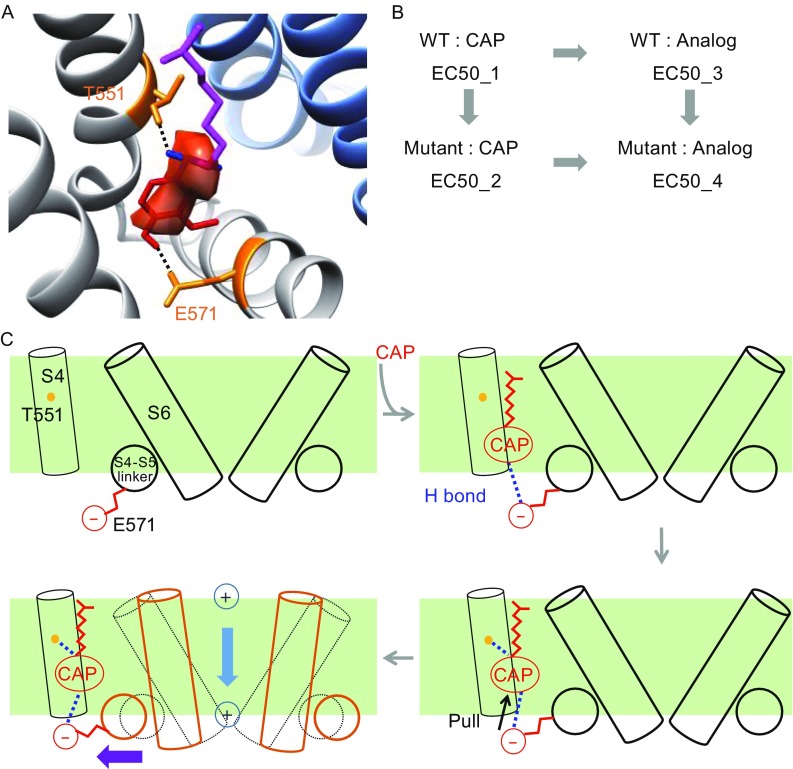
**Mechanism of TRPV1 activation by capsaicin**. (A) Docking of capsaicin into its binding pocket on open-state TRPV1 structure (PDB ID: 3J5R). The Head, Neck and Tail of capsaicin are colored in red, blue and magenta, respectively. Two residues making hydrogen bonds with capsaicin are highlighted in orange. Note that the amino acid numbering for mouse TRPV1 differs from that for rat and human TRPV1 (Fig. 1) by one. (B) Diagram showing the principle of thermodynamic mutant cycle analysis. (C) Cartoon summarizing capsaicin binding and activation of TRPV1

To understand the binding configuration of capsaicin and interactions between capsaicin and TRPV1, a hybrid approach that iteratively combines computational docking and functional studies was developed in 2015 (Yang et al., 2015). As the capsaicin-binding pocket has been well defined by cryo-EM, capsaicin was first computationally docked into this pocket. (To start the docking procedure, capsaicin was put at the entrance of the binding pocket; it robustly found an optimal position inside the binding pocket with the large energy gain from affinity binding.) The chemical environment inside the binding pocket is unique in that it is within the cell membrane, where the energy functions defining atomic interactions are distinct from those in an aqueous environment. Therefore, the membrane energy functions defined in the Rosetta software suite were used to perform the docking (Yarov-Yarovoy et al., 2006; Barth et al., 2007; Leaver-Fay et al., 2011). Indeed, without using membrane-specific energy functions the docking procedure would miss critical hydrogen bonding and therefore yield no good convergence in predictions (Yang et al., 2015). In contrast, with proper membrane energy functions the docking results converged very well, with the head and neck of capsaicin overlapping nicely with the observed electron density of capsaicin. Capsaicin was predicted to take a “tail-up, head-down” configuration within the binding pocket, with its Tail being very flexible to adopt more than one fixed conformations—explaining the lack of clear electron density for the tail in cryo-EM data (Liao et al., 2013; Cao et al., 2013) (Fig. 2A). Upon close scrutiny, such a binding configuration was stabilized by two types of atomic interactions: the van der Waal’s interactions and two hydrogen bonds between its Neck and Head with T551 (T550 in rat and human TRPV1) and E571 (E570 in rat and human TRPV1), respectively (Fig. 2A).

To verify and refine computational predictions, thermodynamic mutant cycle analysis was performed with a series of capsaicin analogs and TRPV1 mutants. This approach has been successfully applied to study interactions between peptide toxin and voltage-gated potassium channels (Ranganathan et al., 1996), as well as between small molecule chemical cAMP and CNG channel (Sunderman and Zagotta, 1999). Briefly, if one part of capsaicin specifically interacts with a residue on the channel, breaking this specific interaction by altering this part of capsaicin or by mutating the interacting residue should have equivalent effects (non-additive). On the other hand, if there is no direct interaction, these changes should have additive effects (Fig. 2B). Specific interaction is assumed only when the calculated coupling energy, kT·ln(Ω), is larger than 1.5 kT (Ranganathan et al., 1996; Schreiber and Fersht, 1995). Indeed, coupling energy values larger than the threshold were only observed between capsaicin Neck and T551, as well as between Head and E571. Moreover, using a series of capsaicin analogs with a progressively shortened Tail, it was shown that the Tail makes a substantial contribution to binding by providing non-specific van der Waals’ interactions with the channel. Therefore, docking results were fully supported by functional studies.

With the capsaicin binding configuration and detailed atomic interactions established, the information was further used as constraints to guide the next round of docking. By docking capsaicin to the closed state, or to open states with or without backbone movements, the sequential events underlying capsaicin binding and activating TRPV1 started to emerge. The Tail and Neck of capsaicin were observed to contact the channel first, mediated by van der Waals interaction and hydrogen bond, respectively. Later on, the Head forms a hydrogen bond with E571 on the S4-S5 linker, which stabilizes its outward movement and subsequent opening of the S6 activation gate (Salazar et al., 2009) (Fig. 2C). This “pull-and-contact” sequence of events during capsaicin activation was also supported by interpolated elastic network modeling, which has successfully predicted the temporal sequence of events in ligand-gated ion channels (Zheng and Auerbach, 2011; Puljung et al., 2014; Tekpinar and Zheng, 2010). Building on the foundation of high-resolution cryo-EM structures, the hybrid approach combining computation and functional studies has unveiled the atomic details of capsaicin-TRPV1 interaction, as well as established a framework for the structural mechanism of ligand-induced channel activation.

A similar cryo-EM based hybrid approach was employed in a study of capsaicin and resiniferatoxin (Elokely et al., 2016). The molecules were first docked by the FRED algorithm (McGann, 2012) of the OpenEye suite into the binding pocket defined by TRPV1 cryo-EM structures, with the constraints of experimentally derived electron density maps of the two ligands. Important residues predicted from docking were tested by point mutations and patch-clamp recordings. This study also found that the Head of capsaicin points downward, while the Tail points upward. Interestingly, this study suggested that, based on calculations from SZMAP in the OpenEye suite, the hydrogen bonds between the Neck/T550 and Head/E570 pairs were mediated by water molecules. The docking of resiniferatoxin in this study is in close agreement to the configuration determined later in the TRPV1 cryo-EM structure with a higher resolution (2.9 Å) in nanodiscs (Gao et al., 2016).

The two studies utilizing a hybrid approach of computation and functional studies illustrated its power in the post-structure era. They demonstrated that, when the protein structure and the general location of ligand binding pocket are defined (by cryo-EM or other structural biology approaches), modern computational tools are able to accurately identify correct binding configuration of the ligand. The reliability of docking results can be further boosted when combined with functional studies such as thermodynamic mutant cycle analysis, which provide critical constrains for computation and validation. Therefore, the hybrid of computation and functional studies is expected to be the new standard approach to understand ligand-channel interactions in the post-structure era.

## Validations of the framework for capsaicin-induced TRPV1 activation

Since the cloning of TRPV1 in 1997, nearly two decades of active research has now established the atomic level framework for how capsaicin binds and activates this channel. This framework will undoubtedly be subjected to further tests. Computationally, molecular dynamic (MD) simulation is a widely used technique to study ligand-host interactions. The “tail-up, head-down” configuration of capsaicin was observed in multiple MD simulations (Darre and Domene, 2015; Hanson et al., 2015; Ohbuchi et al., 2016). Moreover, as the binding pocket locates within the membrane (Liao et al., 2013; Cao et al., 2013), capsaicin has to interact with lipid molecules first before entering the pocket. Such ligand-lipid interaction was also studied by MD simulation (Hanson et al., 2015), which showed that capsaicin flipped from the extracellular to intracellular leaflet of the membrane in order to access the binding pocket.

One of the best ways to validate a proposed mechanism is to experimentally test predictions based on that mechanism. Therefore, if the framework for how capsaicin activates TRPV1 is correct, one might be able to use it as guidance to introduce sensitivity to capsaicin and other vanilloids into ion channels insensitive to these molecules. Two research groups have indeed independently achieved this goal. Based on the current knowledge of capsaicin activation, vanilloid sensitivity was successfully transferred into TRPV2 channel (Yang et al., 2016; Zhang et al., 2016). While TRPV2 is a close homolog of TRPV1, they share only 43% sequence identity between the transmembrane segments surrounding the capsaicin-binding pocket in TRPV1 and the corresponding regions of TRPV2. Impressively, only four point mutations at key residues were needed to introduce a vanilloid-binding site into TRPV2 (Yang et al., 2016; Zhang et al., 2016). Furthermore, the mutant TRPV2 channels were able to bind capsaicin and resiniferatoxin with micromolar apparent affinity. This feat lends a strong support for the current model of capsaicin activation.

## Conclusions and outlook

Elucidation of the mechanistic framework for capsaicin-induced TRPV1 activation is an exciting successful story of modern biomedical research, a story of multidisciplinary investigation driven by rapid technological advancements. Yet, more work is needed in the future to fully understand how capsaicin unlocks the activation machinery of TRPV1. For instance, the kinetics of capsaicin partitioning into the membrane and binding to its pocket on TRPV1 are still poorly defined. Moreover, TRPV1 has been proposed to have two gates, formed by the selectivity filter (the upper gate) and S6 (the lower gate) (Liao et al., 2013; Cao et al., 2013). If so, capsaicin and other TRPV1 activators have to open both gates to initiate ion permeation. The sequential events upon capsaicin binding that lead to channel activation remain to be delineated.

Being a polymodal receptor of noxious stimuli such as capsaicin, proton and high temperature, TRPV1 serves as an important pain sensor (Tominaga et al., 1998; Tominaga and Julius, 2000; Julius, 2013). Many small molecule inhibitors of TRPV1 have been developed by pharmaceutical companies as potential analgesics (Lazar et al., 2009; Moran et al., 2011). However, most of them have failed in clinical trials due to severe side effects such as causing hyperthermia and altering heat sensation in experimental animals and human patients (Carnevale and Rohacs, 2016). Therefore, the knowledge we have gained on how capsaicin activates this channel may lay the foundation for developing novel analgesics targeting TRPV1 without adverse effects. For instance, we have learnt that capsaicin binding induces outward movements of S4-S5 linker (Yang et al., 2015; Liao et al., 2013; Cao et al., 2013), but it induces little conformational changes in the outer pore region as observed in both cryo-EM structures (Liao et al., 2013; Cao et al., 2013) and our FRET studies (Yang et al., 2010). In contrast, large conformational changes occur during heat activation (Yang et al., 2010). Such a difference in movements of the outer pore region may be exploited to develop modality-specific inhibitor of TRPV1 without adverse effects on body temperature and heat sensation.
